# Supplementary material for: Sex-specific multi-level 3D genome dynamics in the mouse brain
Source: Nat Commun. 2022 Jun 15;13:3438. doi: 10.1038/s41467-022-30961-w (PMC9200740; doi:10.1038/s41467-022-30961-w)
Supplement: Supplementary file 1 — Supplementary Information [file 41467_2022_30961_MOESM1_ESM.pdf]

Supplementary Information

Sex-specific multi-level 3D genome dynamics in the mouse brain

Devin Rocks[1†], Mamta Shukla[2†], Laila Ouldibbat[1], Silvia C. Finnemann[1], Achyuth Kalluchi[2], M. Jordan Rowley[2*], Marija Kundakovic[1*]

*Correspondence to: mkundakovic@fordham.edu (M.K.); jordan.rowley@unmc.edu (M.J.R)

Table of Contents

Supplementary Figures

Supplementary Figure 1. Multi-level 3D genome organization in vHIP neurons

Supplementary Figure 2. Compartmental organization in vHIP neurons across sex and the oestrous cycle

Supplementary Figure 3. X chromosome compartmental differences are brain-region specific

Supplementary Figure 4. X chromosome compartmental differences are associated with sex-specific gene expression and motif enrichment

Supplementary Figure 5. CTCF loops in vHIP neurons across sex and the oestrous cycle

Supplementary Figure 6. E-P interactions in vHIP neurons across sex and the oestrous cycle

Supplementary Figure 7. Sex-specific E-P interactions are associated with gene expression in vHIP neurons

Supplementary Figure 8. Sex-specific pathway enrichment of differential E-P interactions

Supplementary Figure 9. 3D genome organization and behavioral response to oestradiol differ between ovary-intact and ovariectomized females

Supplementary Figure 10. Separation of neuronal nuclei using fluorescence-activated nuclei sorting (FANS)

Supplementary Figure 1

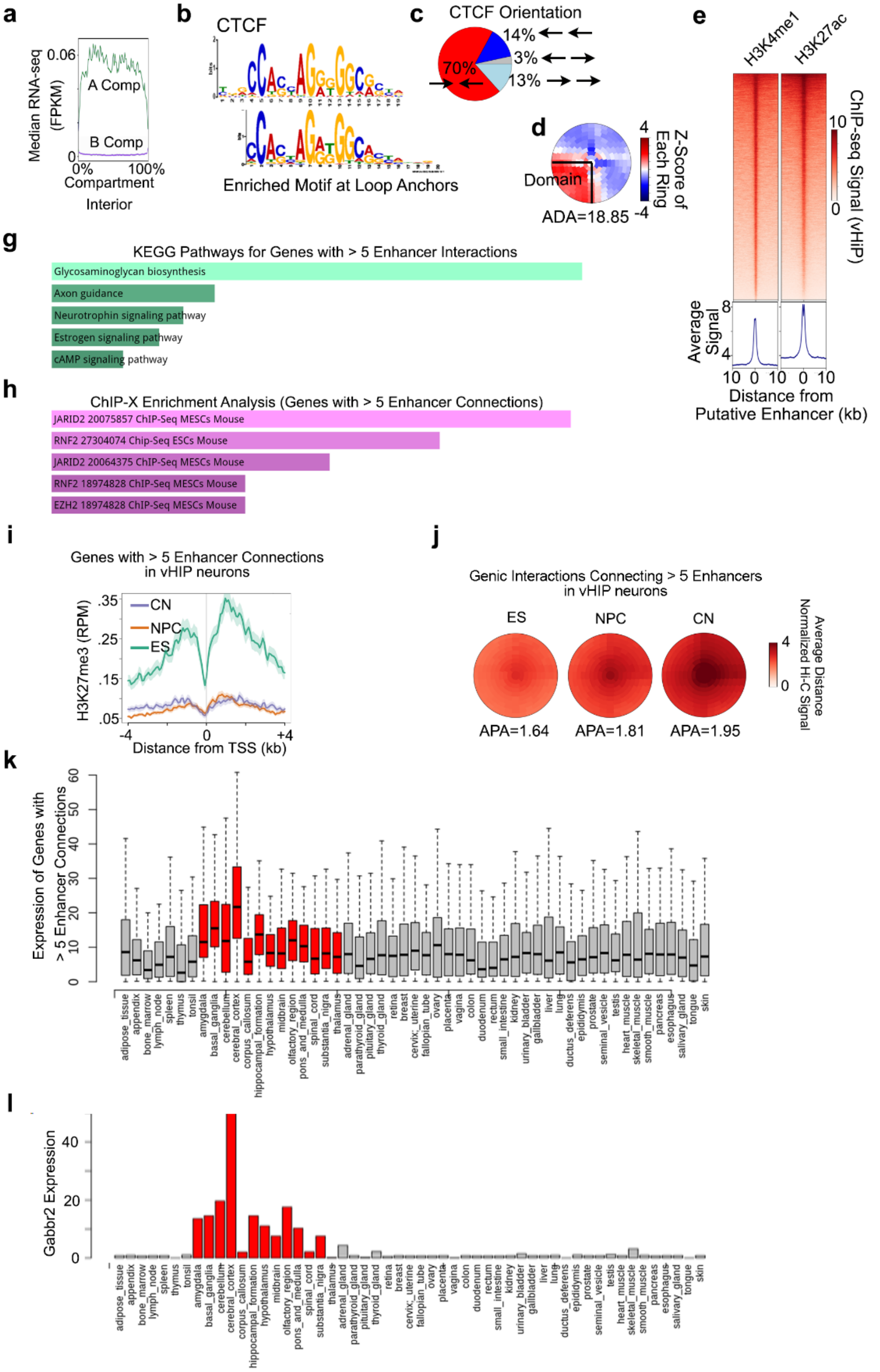

Multi-level 3D genome organization in vHIP neurons. The A chromosomal compartment is associated with active transcription (strong RNA-seq signal) while the B compartment is

associated with transcriptionally inactive genes (**a**). Anchor regions of CTCF loops called by SIP are enriched for the CTCF motif (**b**). CTCF motifs are mostly in the convergent orientation (**c**) and CTCF loops create domains of 3D interactions (**d**). The ATAC-seq peaks used as proxies for enhancers to call enhancer-promoter (E-P) interactions in our study are enriched for H3K4me1 and H3K27ac marks in hippocampal neurons (**e**). Average intensity of promoter-promoter (P-P) and enhancer-enhancer (E-E) interactions are shown in APA plots (**f**). Genes with more than 5 E-P interactions in vHIP neurons are involved in neuronal function and hormone signaling as shown by the KEGG pathway analysis (**g**) and are enriched for binding of repressive polycomb proteins in mouse embryonic stem cells (ESCs) as shown by ChIP-X enrichment analysis (**h**). The same genes are enriched for the repressive histone modification H3K27me3 in embryonic stem (ES) cells, but not in neural progenitor cells (NPC) or cortical neurons (CN; shaded area, standard error) (**i**) and the observed E-P interactions become stronger over the course of neuronal differentiation (**j**). The genes with over 5 E-P interactions (n=130) in vHIP neurons are primarily expressed in the central nervous system. Box plots (box, 1st-3rd quartile; horizontal line, median; whiskers, 1.5x IQR) (**k**). These genes include *Gabbr2*, encoding a subunit of the GABA-B receptor (**l**). Data by Halder et al[32] generated using sorted hippocampal neurons were used to test the enrichment of H3K4me1 and H3K27ac marks in our non-promoter ATAC-seq peaks. Data by Bonev et al.[18] were used to examine H3K27me3 levels and E-P interactions in our multi-enhancer genes during neuronal differentiation. Data by Uhlen et al.[34] were used to test the expression of these genes across tissues.

Supplementary Figure 2

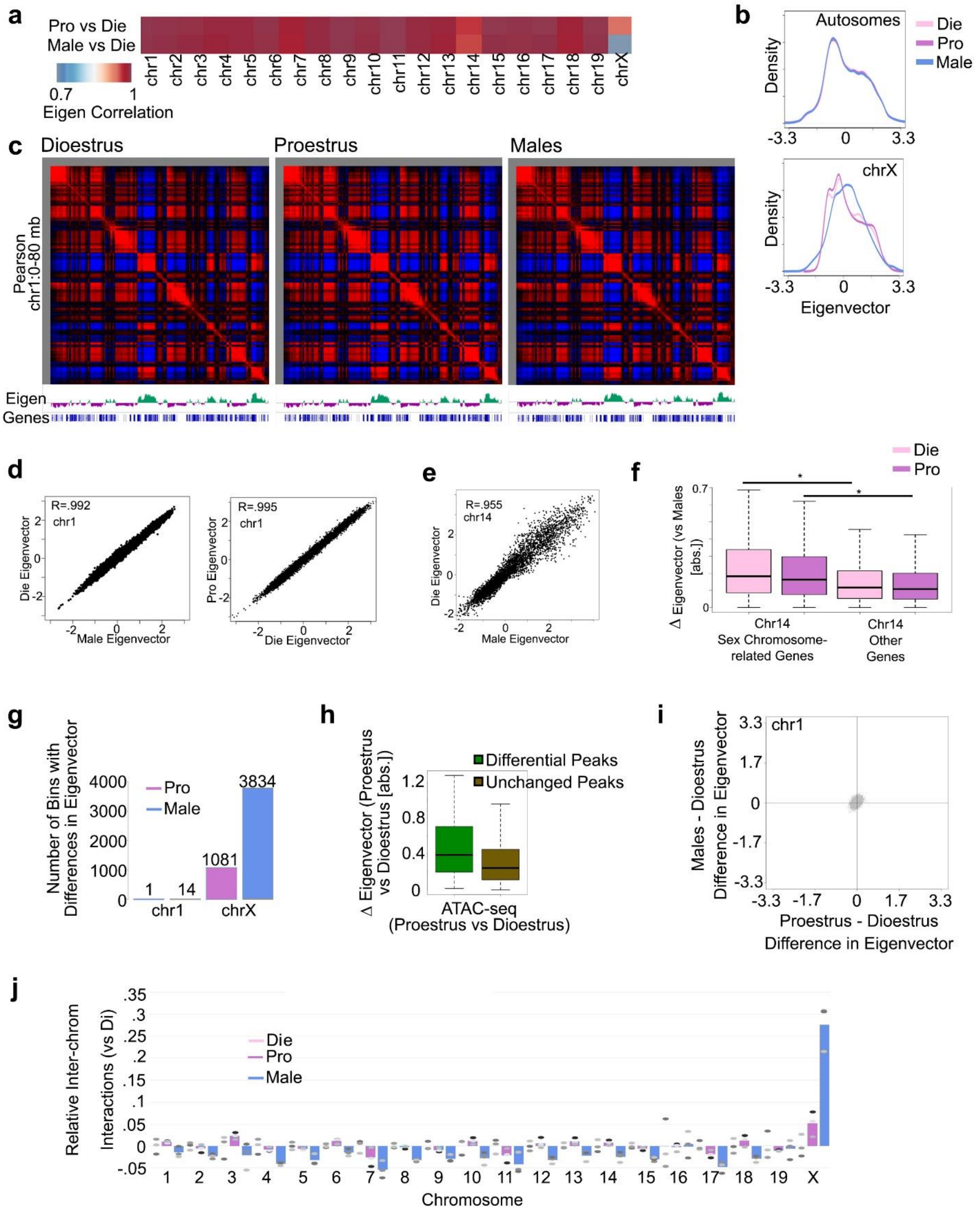

Compartmental organization in vHIP neurons across sex and the oestrous cycle. Sex- and oestrous cycle-dependent compartmental changes, displayed by eigenvector heatmaps (**a**) and

density plots (**b**) are primarily observed on the X chromosome. A correlational Hi-C matrix with eigenvector signal (Eigen) for an 80 Mb-region of the chromosome 1 (**c**) shows a high correlation of compartmental signal in Die-Male and Die-Pro comparisons (**d**). Chromosome 14 exhibits compartmental changes (**e**) that are enriched in Chr14 genes previously demonstrated to undergo chromatin regulation similar to that of the sex chromosomes (n=325 sex chromosome-related genes; n=4,460 remaining chr14 genes); Wilcoxon rank-sum test; *, $P<0.05$ (**f**). The number of 25-kb bins with differences in eigenvector across-sex and within-females for the X chromosome and chromosome 1 (**g**). X chromosome bins with differences in eigenvector within-females also display differential chromatin accessibility (n=24 differential peaks, n=1,481 non-differential peaks) (**h**). No association was observed between Pro-Die and Die-Male compartmental changes on the chromosome 1 (**i**). When each separate biological replicate was examined (see differentially colored ellipses), proestrus and males display a greater number of inter-chromosomal interactions on the X-chromosome, but not on autosomes, compared to dioestrus (**j**). Box plots (box, 1st-3rd quartile; horizontal line, median; whiskers, 1.5x IQR).

Supplementary Figure 3

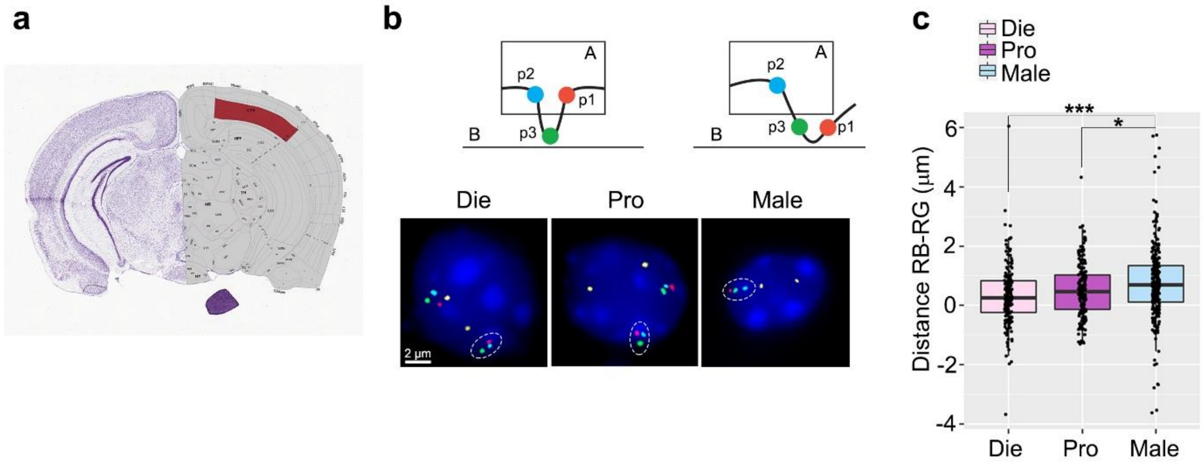

X chromosome compartmental differences are brain region-specific. FISH analysis was performed in the visual cortex, highlighted in (**a**). The compartmental change identified in the vHIP (**Fig. 2e-g**, a switch from compartment A to B in Die vs. Pro) was not found in the visual cortex, rather we identified a sex difference between males and both female groups (representative images are shown; n=3 animals/group; scale bar: 2 µm; note: yellow signal is from a control probe on chromosome 1) (**b**). FISH data were calculated as the RB-RG distance by subtracting the distance between the center of the red and green signals (RG) from that of the red and blue signals (RB). The analysis was restricted to X chromosomes positive for all three probe signals (n=190 Die; n=194 Pro; n=244 Male); Box plots (box, 1st-3rd quartile; horizontal line, median; whiskers, 1.5x IQR); one-way ANOVA with post-hoc Tukey; *, P<0.05 (P=0.0387); ***, P<0.001 (P=0.000143). Source data are provided as a Source Data file (**c**).

Supplementary Figure 4

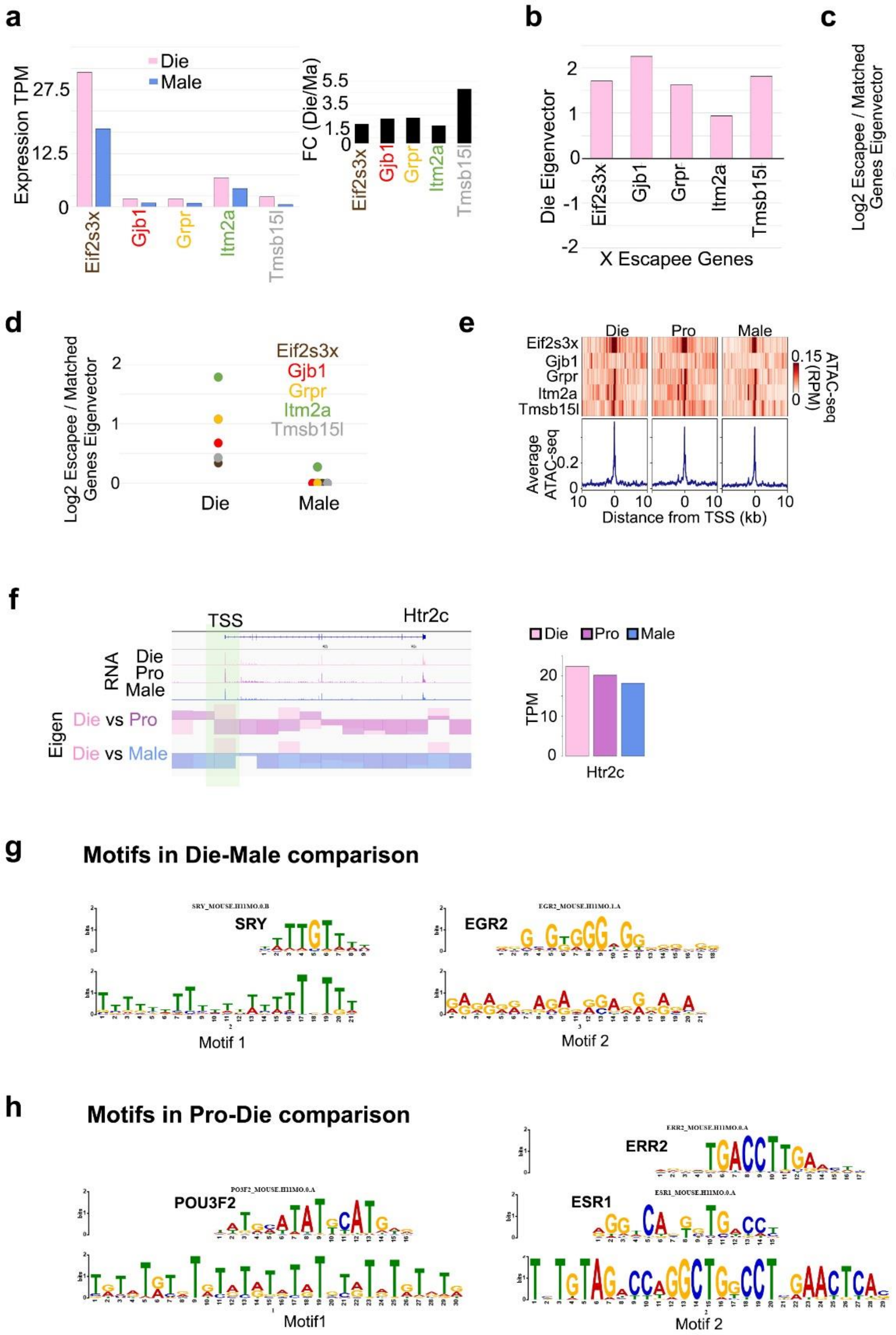

X chromosome compartmental differences are associated with sex-specific gene expression and motif enrichment. Expression levels (left) and expression fold-change (right) of five X-linked genes in the Die-Male comparison (**a**), along with their A compartmental profiles in

dioestrus (**b**) indicate these genes undergo X-escape. Relative to genes with similar expression levels (n=400), expression of these escapees (n=5) is associated with a higher eigenvector signal in dioestrus compared to males on average (error bars, SEM) (**c**) and by individual escapee (**d**). The promoters of escapee genes do not show differences in ATAC-seq signal (**e**), indicating compartmental interactions as a better predictor of X escape. Eigenvector tracks for *Htr2c* (left) indicate differential compartmental profiles across the oestrous cycle and sex that overlap the transcription start site (TSS) and correlate with altered expression levels (right, **f**). Note that we previously identified *Htr2c* as a variable X escapee in adult vHIP neurons, which is differentially expressed in the Die-Male but not in the Pro-Male comparison[14]. Motif analyses of genomic regions with differential compartment signals between groups show enrichment for Sry (E-value 7.3e-75) and Egr2 (E-value 3.7e-66) binding sites in the Die-Male comparison (**g**), and enrichment for Pou3f2 (E-value 1.7e-100) as well as ERα and ERR2 (E-value 2.0e-67) binding sites in the Pro-Die comparison (**h**).

Supplementary Figure 5

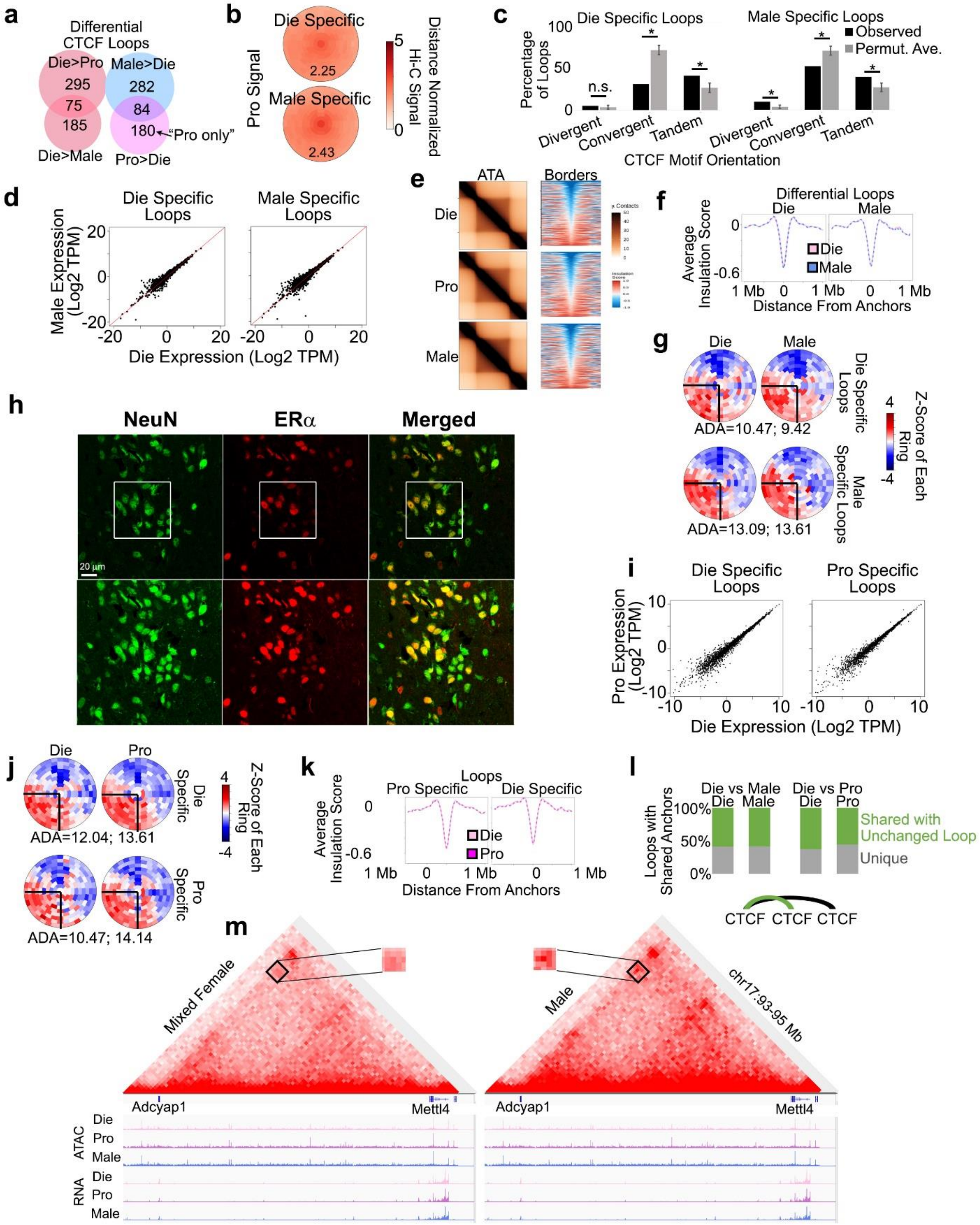

CTCF loops in vHIP neurons across sex and the oestrous cycle. A venn diagram of differential loops between all samples illustrates the specificity of differential loops to each comparison (**a**). The loop signal of proestrus females is between that of diestrus and males (**b**). CTCF motifs at differential loops are frequently in non-convergent orientations (n=1000 permutations each; error bars, standard deviation); Monte Carlo permutation test; *, $P<0.05$ (**c**). On a global level, gene expression in dioestrus and males is similar whether the gene is within a dioestrus- or male-specific loop (**d**). Aggregate TAD Analysis and insulation scores at borders showed minimal differences between samples (**e**). Differential loops were not associated with differences in insulation scores (**f**) or interactions domains (**g**). Confocal microscopy images of vHIP tissue sections stained for NeuN, a marker for neuronal nuclei, and ERα, with an overlap indicating ERα is present in vHIP neuronal nuclei, consistent with its possible role as a loop organizer in these cells (**h**). The same field is shown as x-y section (upper row) and the maximum intensity projection (lower row); the smaller, boxed region was shown in the main Figure 4e. While the image shown depicts a proestrus sample, nuclear ERα was observed in NeuN+ cells of all three groups (n=3 animals/group; scale bar: 20 μm, **h**). On a global level, gene expression in proestrus and dioestrus is similar whether the gene is within a proestrus- or dioestrus-specific loop (**i**). Pro-Die differential loops were not associated with differences in interaction domains (**j**) or insulation scores (**k**), and most of the differential loops in both Pro-Die and Die-Male comparisons share an anchor with an unchanged loop (**l**). A specific, 2Mb-loop connecting *Adcyap1* and an upstream region of *Mettl4* found to be stronger in proestrus than in dioestrus (**Fig. 4f**), is identified as a differential loop in merged-females compared to males, with a weaker signal in females, emphasizing a loss of specificity when the female groups are merged (**m**).

Supplementary Figure 6

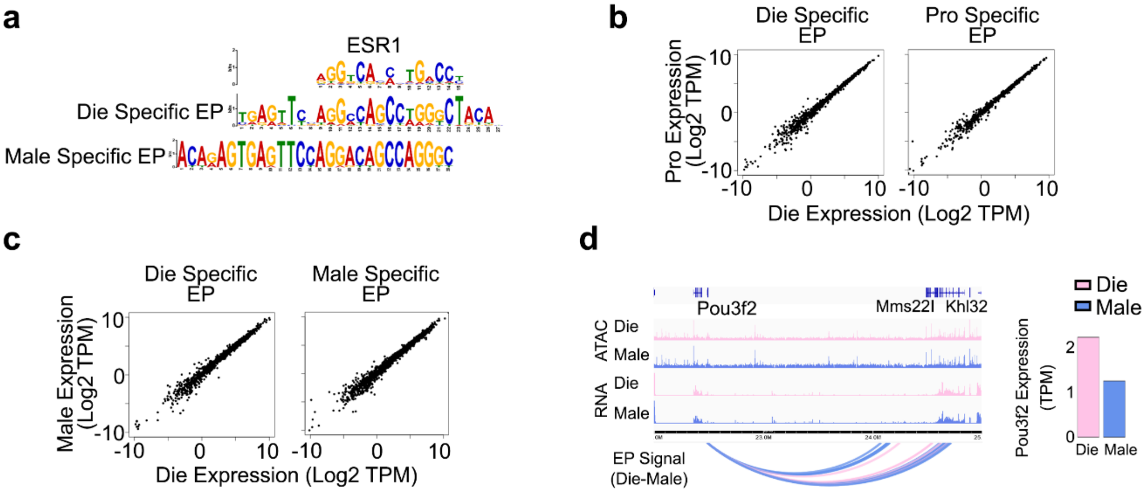

E-P interactions in vHIP neurons across sex and the oestrous cycle. Genomic regions with differential enhancer-promoter (E-P) interactions in the Die-Male comparison are enriched with ERα binding sites (E-values of 2.9e-100 and 6.5e-106) (**a**). Gene expression within-females is similar whether the gene has Die- or Pro-specific E-P interactions (**b**), and gene expression across sex is similar whether the gene has Die- or Male-specific E-P interactions (**c**). *Pou3f2* exhibits differential E-P interactions in the Die-Male comparison that are associated with differential gene expression (**d**). IGV tracks show merged ATAC-seq and RNA-seq data for dioestrus (Die), and males (Male), and the bar graph (on the right) shows *Pou3f2* gene expression. All data were derived from 3 biological replicates for each group.

Supplementary Figure 7

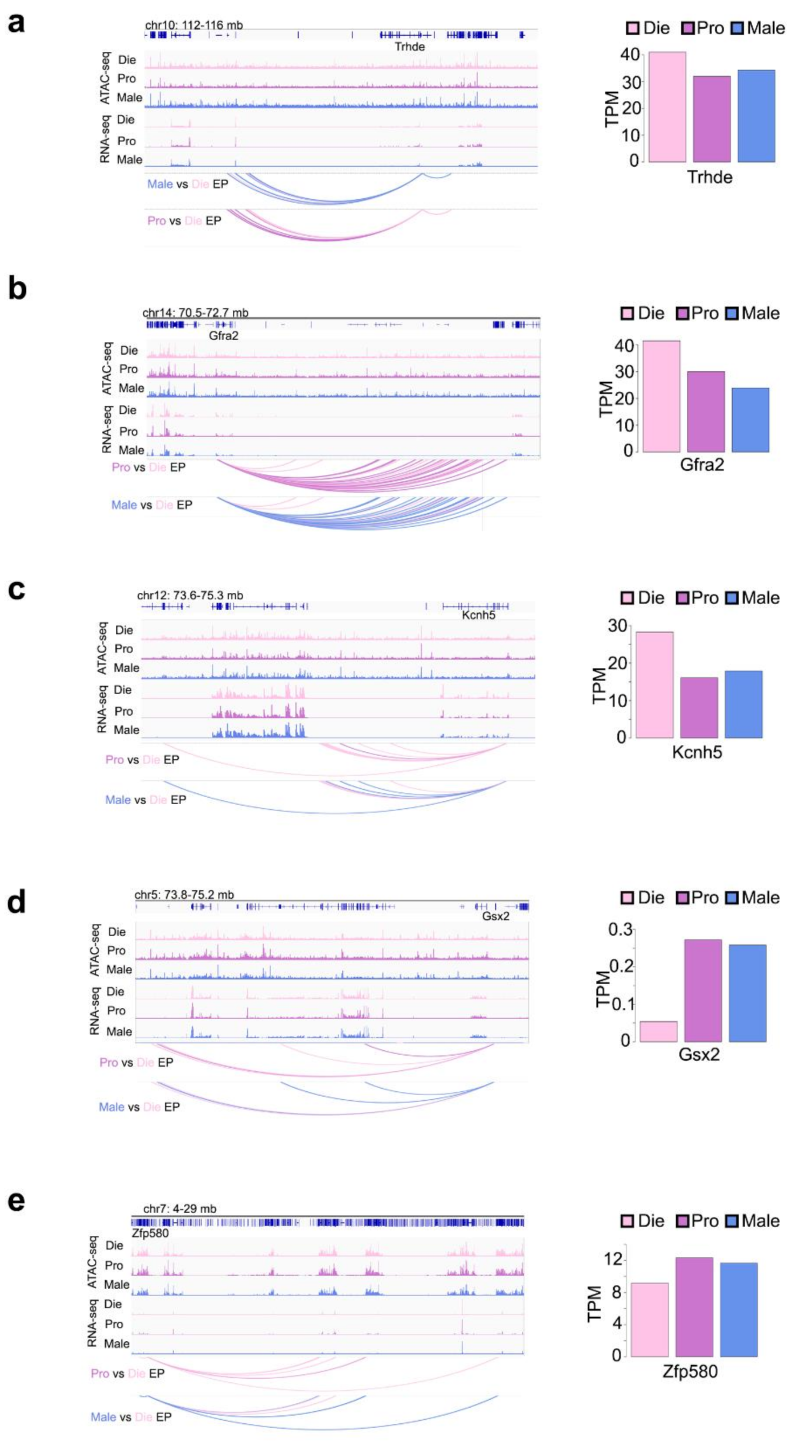

Sex-specific E-P interactions are associated with gene expression in vHIP neurons. Example genes showing differential E-P interaction profiles and differential gene expression dependent on

sex and oestrous cycle stage including *Trhde* (**a**), *Gfra2* (**b**), *Kcnh5* (**c**), *Gsx2* (**d**), and *Zfp580* (**e**). IGV tracks show merged ATAC-seq and RNA-seq data for dioestrus (Die), proestrus (Pro), and males (Male) derived from 3 biological replicates for each group. Bar graphs (on the right) show gene expression for each gene. Die, pink; Pro, purple; Male, blue.

Supplementary Figure 8

a

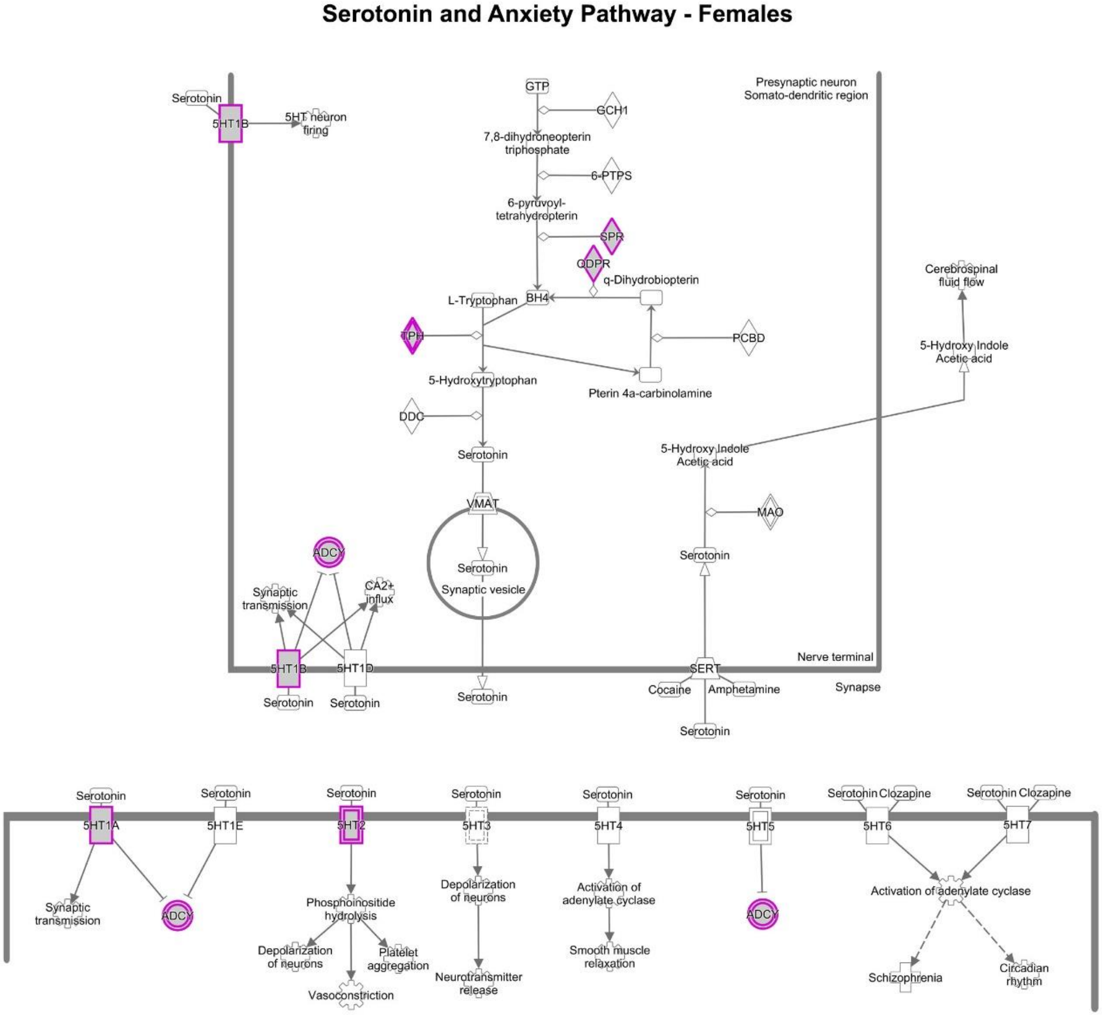

b

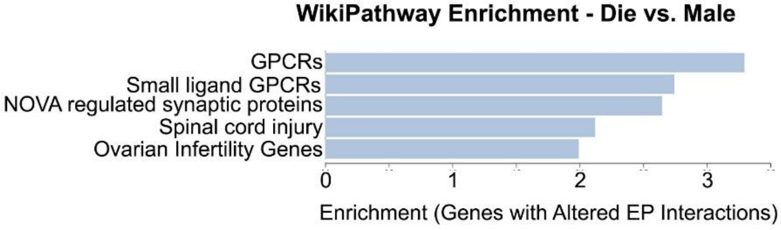

Sex-specific pathway enrichment of differential E-P interactions. (**a**) WikiPathway schematic of the Serotonin and Anxiety pathway. Proteins with purple borders are encoded by genes with differential E-P interactions within-females across the oestrous cycle. (**b**) In the Die-Male comparison, top enriched pathways of differential E-P interactions are involved in G protein-coupled receptor (GPCR) signaling.

Supplementary Figure 9

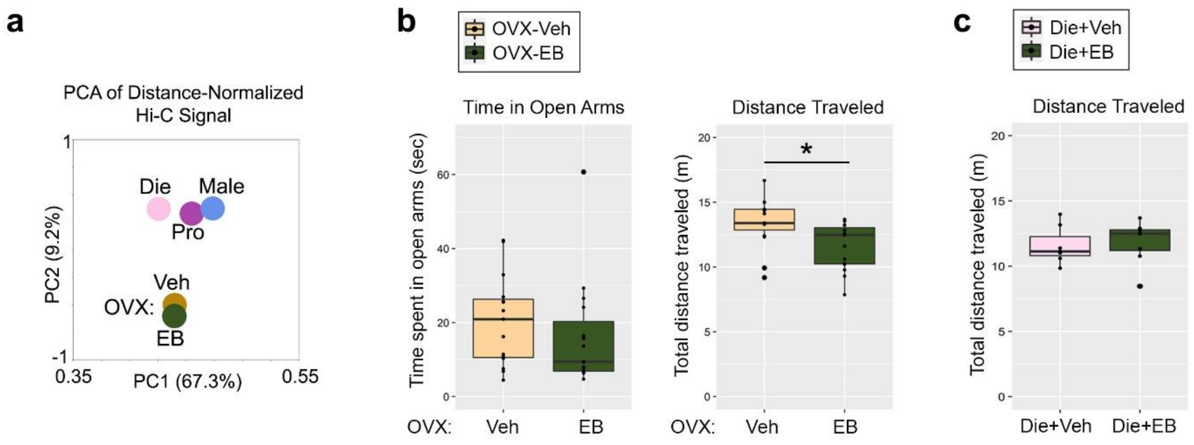

3D genome organization and behavioral response to oestradiol differ between ovary-intact and ovariectomized females. A PCA plot of all Hi-C samples following downsampling indicates an overall difference in Hi-C signal between ovariectomized (OVX) females and ovary-intact (dioestrus, Die; proestrus, Pro) females (**a).** In the elevated plus maze (box plots), an acute oestradiol benzoate (EB) treatment did not change time spent in the open arms (left) but it decreased overall activity levels (P=0.0112, right) in OVX females (n=15 animals/group, **b**). In ovary-intact dioestrus (Die) females, EB treatment did not affect overall activity levels (n=7 Die+Veh, n=8 Die+EB, **c**). Box plots (box, 1st-3rd quartile; horizontal line, median; whiskers, 1.5x IQR); paired-sample two-sided T-test; *, P<0.05. Source data are provided for the graphs in (**b**) and (**c**) as a Source Data file. OVX-Vehicle, yellow; EB, green; Die, pink; Pro, purple; Male, blue.

Supplementary Figure 10

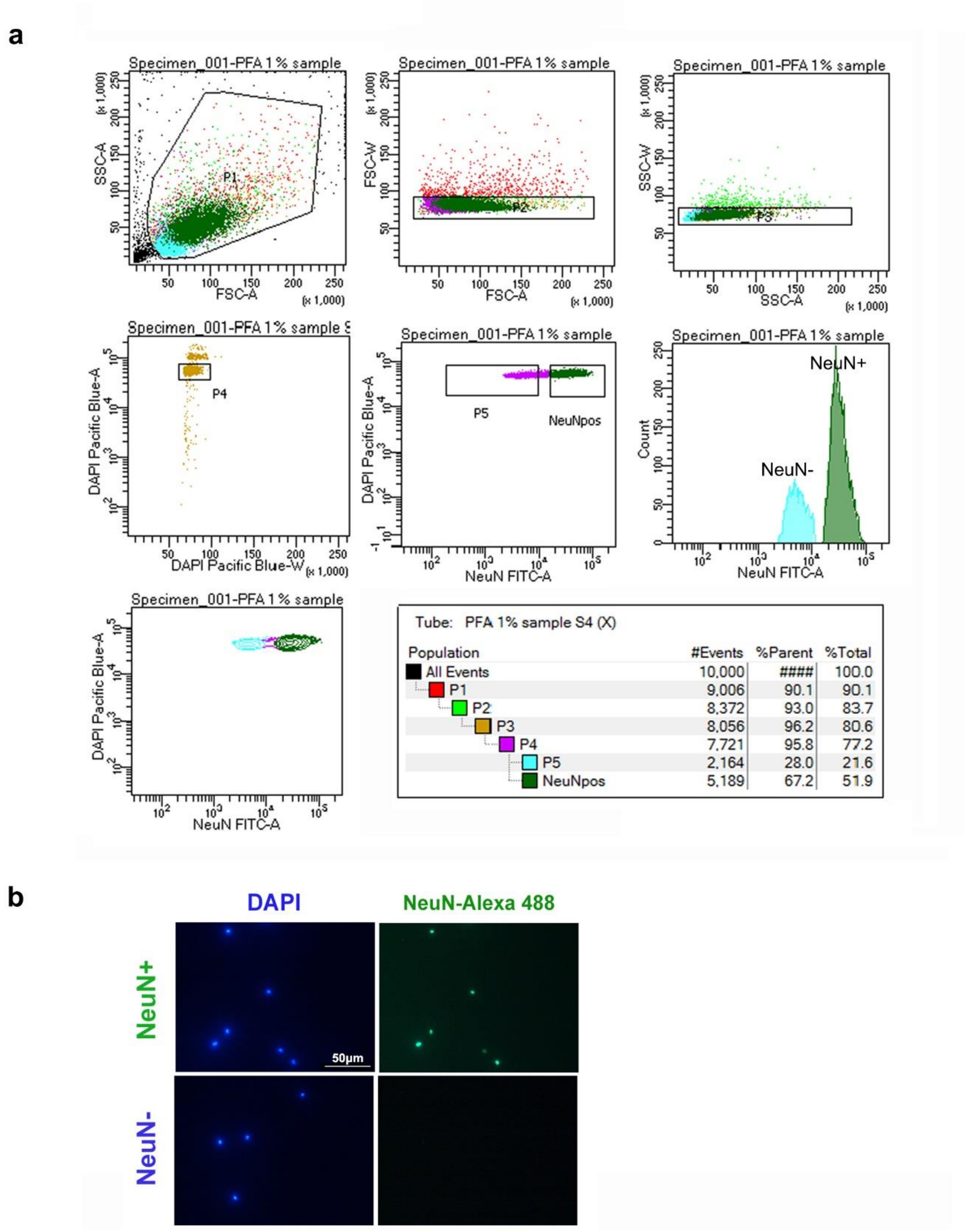

Separation of neuronal nuclei using fluorescence-activated nuclei sorting (FANS). A representative FANS report shows the gating strategy that was used to: 1) separate nuclei from debris (P1-P3); 2) ensure the sorting of single nuclei using the DAPI signal (P4); and 3) select the

NeuN+ neuronal nuclei (P6) from the NeuN- non-neuronal nuclei (P5) (**a**). Representative immunofluorescence microscopy images attained after sorting demonstrate that sorting separates NeuN+ from NeuN- nuclei and results in a single-nuclei suspension (scale bar: 50 μm; **b**). We have performed this microscopy quality assessment 3 times with identical results.
