# Supplementary material for: Sex-specific multi-level 3D genome dynamics in the mouse brain
Source: Nat Commun. 2022 Jun 15;13:3438. doi: 10.1038/s41467-022-30961-w (PMC9200740; doi:10.1038/s41467-022-30961-w)
Supplement: Supplementary file 3 — Description of additional Supplementary File [file 41467_2022_30961_MOESM3_ESM.pdf]

Descriptions of Additional Supplementary Data Files

Supplementary Data 1: Hi-C data basic information for the oestrous cycle experiment

Supplementary Data 2: List of genes with multiple E-P interactions

Supplementary Data 3: List of genes associated with differential loops and E-P interactions across the oestrous cycle

Supplementary Data 4: Hi-C data basic information for the oestrogen replacement experiment

Supplementary Data 5: Genes overlapping differential E-P interactions in Die-Pro and EB-Vehicle comparisons
