# Supplementary material for: Sex-specific multi-level 3D genome dynamics in the mouse brain
Source: Nat Commun. 2022 Jun 15;13:3438. doi: 10.1038/s41467-022-30961-w (PMC9200740; doi:10.1038/s41467-022-30961-w)
Supplement: Supplementary file 9 — Reporting Summary [file 41467_2022_30961_MOESM9_ESM.pdf]

nature portfolio

Corresponding author(s): Marija Kundakovic, M. Jordan Rowley

Last updated by author(s): May 8, 2022

Reporting Summary

Nature Portfolio wishes to improve the reproducibility of the work that we publish. This form provides structure for consistency and transparency in reporting. For further information on Nature Portfolio policies, see our Editorial Policies and the Editorial Policy Checklist.

Statistics

For all statistical analyses, confirm that the following items are present in the figure legend, table legend, main text, or Methods section.

n/a	Confirmed	
☐	☒	The exact sample size (*n*) for each experimental group/condition, given as a discrete number and unit of measurement
☐	☒	A statement on whether measurements were taken from distinct samples or whether the same sample was measured repeatedly
☐	☒	The statistical test(s) used AND whether they are one- or two-sided<br>*Only common tests should be described solely by name; describe more complex techniques in the Methods section.*
☒	☐	A description of all covariates tested
☐	☒	A description of any assumptions or corrections, such as tests of normality and adjustment for multiple comparisons
☐	☒	A full description of the statistical parameters including central tendency (e.g. means) or other basic estimates (e.g. regression coefficient) AND variation (e.g. standard deviation) or associated estimates of uncertainty (e.g. confidence intervals)
☐	☒	For null hypothesis testing, the test statistic (e.g. *F*, *t*, *r*) with confidence intervals, effect sizes, degrees of freedom and *P* value noted<br>*Give P values as exact values whenever suitable.*
☒	☐	For Bayesian analysis, information on the choice of priors and Markov chain Monte Carlo settings
☒	☐	For hierarchical and complex designs, identification of the appropriate level for tests and full reporting of outcomes
☐	☒	Estimates of effect sizes (e.g. Cohen's *d*, Pearson's *r*), indicating how they were calculated

Our web collection on statistics for biologists contains articles on many of the points above.

Software and code

Policy information about availability of computer code

Data collection	Immunofluorescence data were processed using Leica confocal LAS AF software (Leive Microsystems GmbH). Flow cytometry data were collected using BD FACSDiva v8.0.1 software. High-performance Liquid Chromatography with Tandem Mass Spectometry (HPLC/MS-MS) data were collected using MassHunter Workstation Data Acquisition for Triple Quad B.07.01/Build 7.1.7112.0.
Data analysis	Flow cytometry data were analyzed using BD FACSDiva v8.0.1 software. HPLC/MS-MS data were analyzed using MassHunter Workstation Data Acquisition for Triple Quad B.07.01/Build 7.1.7112.0.<br>We used the following packages and software for bioinformatic analysis:<br>R (https://cran.r-project.org/); BWA (http://bio-bwa.sourceforge.net/); DESeq2 (https://bioconductor.org/packages/release/bioc/html/DESeq2.html); Juicebox (https://aidenlab.org/juicebox/); SIP (https://github.com/PouletAxel/SIP); FitHiC2(https://ay-lab.github.io/fithic/); SIPMeta (https://github.com/PouletAxel/SIPMeta); ngs.plot (https://github.com/shenlab-sinai/ngsplot); meme-chip (https://meme-suite.org/meme/tools/meme-chip); Stringtie (https://ccb.jhu.edu/software/stringtie/); EnrichR (https://cran.r-project.org/web/packages/enrichR/vignettes/enrichR.html); Ingenuity Pathway Analysis (https://www.qiagenbioinformatics.com/products/ingenuitypathway-analysis); GENOVA (https://github.com/robinweide/GENOVA).<br>Distance between FISH probes was analyzed using ImageJ v1.53 (public domain software from the National Institutes of Health; http://imagej.nih.gov/ij/).

For manuscripts utilizing custom algorithms or software that are central to the research but not yet described in published literature, software must be made available to editors and reviewers. We strongly encourage code deposition in a community repository (e.g. GitHub). See the Nature Portfolio guidelines for submitting code & software for further information.

Data

Policy information about availability of data

All manuscripts must include a data availability statement. This statement should provide the following information, where applicable:

- Accession codes, unique identifiers, or web links for publicly available datasets
- A description of any restrictions on data availability
- For clinical datasets or third party data, please ensure that the statement adheres to our policy

Hi-C data are available from the NCBI Gene Expression Omnibus (GEO) database under accession number GSE172228.
ATAC-seq and nuclear RNA-seq data on sorted vHIP neurons from diestrus, proestrus and male groups were previously generated in triplicates (14) and are available from the GEO database under accession number GSE114036.
The H3K4me1 and H3K27ac ChIP-seq data used to validate enhancers is available from the GEO database under accession numbers GSM1939123 and GSM1939156, respectively.
The HiC and H3K27me3 ChIP-seq data from ES, NPC, and CN is available from GEO accession GSE96107 and from ENCODE accession ENCSR059MBO.
The mm10 genome assembly is available at https://www.ncbi.nlm.nih.gov/assembly/GCF_000001635.20/.
Source data are provided with this paper.

Field-specific reporting

Please select the one below that is the best fit for your research. If you are not sure, read the appropriate sections before making your selection.

☒ Life sciences ☐ Behavioural & social sciences ☐ Ecological, evolutionary & environmental sciences

For a reference copy of the document with all sections, see nature.com/documents/nr-reporting-summary-flat.pdf

Life sciences study design

All studies must disclose on these points even when the disclosure is negative.

Sample size	No statistical methods were used to determine sample size. Sample sizes were chosen based on previous experience and published work (PMID:31253786, PMID:22955991). The oestrous cycle Hi-C experiment included 3 biological replicates (each pooled from 2 animals) per group, while the OVX-oestradiol replacement Hi-C experiments included 5 biological replicates (pooled from 2 animals each) per group. The FISH experiment included 3 animals or 116-163 chromosomes per group for the ventral hippocampus, and 3 animals or 190-244 chromosomes per group for the visual cortex. The confocal analysis included 3 animals per group. Behavioral experiments included 7-15 animals per group.
Data exclusions	For the FISH analysis, probe signals were excluded from measurement if all three X chromosome probe signals were not present in the image.
Replication	Since these were animal studies, we used biological replicates in all experiments. For Hi-C experiments, we used 3 biological replicates (derived from 6 mice) per group for the oestrous cycle study and 5 biological replicates (derived from 10 mice) per group for the oestrogen replacement study; we showed the high correlation between the replicates in each group (Figure 1c). For the FISH analysis, we used 3 animals or 116-163 chromosomes per group for the ventral hippocampus, and 3 animals or 190-244 chromosomes per group for the visual cortex. For confocal microscopy, we used 3 animals per group. For behavioral analyses, we used 7-15 animals per group.
Randomization	We did not perform randomization since our experiments were dependent on natural hormone fluctuations in females. We placed females in two groups (proestrus or dioestrus) based on their cycling patterns and the day of tissue collection was dependent on the duration of their estrous cycles. However, we made sure to have equal group (proestrus, diestrus, and males) distributions in each batch (e.g. for tissue collection, sorting, library prep, sequencing), whenever possible.
Blinding	Blinding was not a possibility in this study because we used estrous cycle stage predictions for females and had to predetermine which day and time of day to perform tissue collection for each animal based on these predictions. For all experiments and sample processing that were performed on separate days, samples were equally distributed to control for batch effects.

Reporting for specific materials, systems and methods

We require information from authors about some types of materials, experimental systems and methods used in many studies. Here, indicate whether each material, system or method listed is relevant to your study. If you are not sure if a list item applies to your research, read the appropriate section before selecting a response.

Materials & experimental systems

n/a	Involved in the study
☐	☒ Antibodies
☒	☐ Eukaryotic cell lines
☒	☐ Palaeontology and archaeology
☐	☒ Animals and other organisms
☒	☐ Human research participants
☒	☐ Clinical data
☒	☐ Dual use research of concern

Methods

n/a	Involved in the study
☒	☐ ChIP-seq
☐	☒ Flow cytometry
☒	☐ MRI-based neuroimaging

Antibodies

Antibodies used	Antibodies for FACS: Mouse monoclonal Anti-NeuN Antibody, clone A60, conjugated to AlexaFluor 488 (MAB377X; Millipore); and Mouse monoclonal IgG1-k, clone MOPC-21 antibody control, conjugated to Alexa Fluor 488 (FCMAB310A4, Millipore).<br>Primary antibody for immunofluorescence analysis: rabbit polyclonal anti-ERα antibody (Sigma-Aldrich, 06-935).<br>Secondary antibody for immunofluorescence analysis: donkey anti-rabbit IgG conjugated to AlexaFluor-594 (Invitrogen, A-21207).
Validation	Mouse monoclonal anti-NeuN antibody conjugated to AlexaFluor 488 (MAB377X; Millipore) - previously validated for fluorescence-activated nuclei sorting (PMID:27113501, PMID:31253786) and confirmed by immunofluorescence in this study.<br>Mouse monoclonal IgG1-k, clone MOPC-21 antibody control, conjugated to Alexa Fluor 488 (FCMAB310A4, Millipore) - validated by the manufacturer, whose product page states: "Mouse IgG1-k Monoclonal Antibody control validated for use in Flow Cytometry."; "This mouse IgG1 isotype control antibody is not reactive with any known antigen." We have also internally validated this antibody as a negative control for our FACS sorting (PMID:31253786).<br>Rabbit polyclonal anti-ERα antibody (Sigma-Aldrich, 06-935) - validated by Western Blot in MCF7 cell lysates by the manufacturer; by Western blot in ventral hippocampal cell lysate; and by immunofluorescence in several publications (e.g. PMID: 24646567, PMID: 25157819, PMID: 25324747).<br>Donkey anti-rabbit IgG conjugated to AlexaFluor-594 (Invitrogen, A-21207) - verified for immunohistochemistry by the manufacturer and several publications (e.g. PMID: 27197019)

Animals and other organisms

Policy information about studies involving animals; ARRIVE guidelines recommended for reporting animal research

Laboratory animals	For the oestrous cycle experiments, male and female C57BL/6J mice from Jackson Laboratory arrived at seven weeks of age. For the oestrogen replacement experiments, females were ovariectomized at 8 weeks and arrived at 9 weeks of age. All animals were housed in same-sex cages (n = 3-5 per cage). Mice were habituated for two weeks and were kept on a 12:12h light:dark cycle (lights on at 8 a.m.) with ad libitum access to food and water. Temperature of the room is set to 21°C and humidity ranges from 30-70%. For oestrous cycle experiments, the oestrous cycle of female animals was tracked daily in the morning (between 9AM and 11AM) for two weeks (between 9 and 11 weeks of age). OVX animals were acutely treated (for 4 hours) with either oestradiol benzoate or vehicle at 11 weeks of age. All male and female animals were sacrificed via cervical dislocation at 11 weeks of age. All animal procedures were approved by the Institutional Animal Care and Use Committee at Fordham University.
Wild animals	This study did not include wild animals
Field-collected samples	This study did not include field-collected samples
Ethics oversight	All animal procedures were approved by the Institutional Animal Care and Use Committee at Fordham University.

Note that full information on the approval of the study protocol must also be provided in the manuscript.

Flow Cytometry

Plots

Confirm that:

☒ The axis labels state the marker and fluorochrome used (e.g. CD4-FITC).

☒ The axis scales are clearly visible. Include numbers along axes only for bottom left plot of group (a 'group' is an analysis of identical markers).

☒ All plots are contour plots with outliers or pseudocolor plots.

☒ A numerical value for number of cells or percentage (with statistics) is provided.

Methodology

Sample preparation	Bilateral ventral hippocampi were dissected from the brain and snap frozen in liquid nitrogen. Total nuclei were then extracted from hippocampi from two animals using ultracentrifugation through a sucrose gradient. Neuronal (NeuN+) nuclei were isolated by fluorescence-activated nuclei sorting with anti-NeuN antibody conjugated to AlexaFluor 488 on a FACSAria

	instrument. A detailed protocol for nuclei isolation and purification of neuronal nuclei is provided in Kundakovic et al, 2017 (PMID:27113501).
Instrument	FACS Aria instrument (BD Sciences, San Jose, CA); Cat No: 337667
Software	BD FACSDiva v8.0.1 software
Cell population abundance	For each Hi-C biological replicate, we collected 200,000 NeuN+ (neuronal) nuclei.
Gating strategy	In addition to a sample containing NeuN-AlexaFluor 488 and DAPI stain, three controls were used to set up the gates for sorting: DAPI only; IgG1 isotype control-AlexaFluor 488 and DAPI; and NeuN-AlexaFluor 488 only. We set up the protocol to remove debris, ensure single nuclear sorting (using DAPI), and select the NeuN+ (neuronal) and NeuN- (non-neuronal) nuclei populations. The gating strategy is presented in Supplementary Figure 10. We have previously described this method in detail (PMID:27113501).

☒ Tick this box to confirm that a figure exemplifying the gating strategy is provided in the Supplementary Information.
